# A single‐center comparative analysis of outpatients with and without COVID‐19 in Sapporo, Japan

**DOI:** 10.1002/jgf2.483

**Published:** 2021-07-16

**Authors:** Yoshinosuke Shimamura, Kaihei Masuda, Yoshiyasu Anbo, Fumiko Sugaya, Yasushi Furuta

**Affiliations:** ^1^ Office of Infection Control and Prevention Teine Keijinkai Medical Center Sapporo Hokkaido Japan

**Keywords:** COVID‐19, Japan, myalgia, reverse transcriptase‐polymerase chain reaction

## Abstract

This study aimed to investigate the demographic, clinical, and epidemiological statistics of Japanese patients attending a designated outpatient clinic for COVID‐19 in Sapporo, Japan, and contrast the clinical and epidemiological features between those with and without mild COVID‐19. A total of 27 (8.6%) of 315 patients were diagnosed with COVID‐19. They had higher proportions of myalgia, direct contact with a confirmed COVID‐19 patient, and attendance of social gatherings in close confines. We believe that our study makes a significant contribution to the literature because it provides a clinical picture of mild COVID‐19 in the Japanese population, which has not been studied extensively. It can also assist in optimizing the local preventive measures to reduce the transmission of COVID‐19.

## 1 INTRODUCTION

Coronavirus disease 2019 (COVID‐19) has become a global pandemic.^1^ The Japanese public health agencies found clustering in the “Three Cs”: closed spaces with poor ventilation, crowded spaces with many people, and close contact^2^; however, the clinical investigation of Japanese patients with COVID‐19 is limited.^3^, ^4^, ^5^, ^6^, ^7^ Besides, no study has existed evaluating COVID‐19 in outpatient settings although understanding COVID‐19 is crucial for frontline clinical practitioners. We aimed to describe and compare the demographic, clinical, and epidemiological characteristics and outcomes of patients with and without COVID‐19 who presented to the Teine Keijinkai Medical Center Infection Triage Clinic, Sapporo, Japan.

## 2 METHODS

We reviewed all clinical records of patients presenting at the hospital's Infection Triage Clinic between February 14, 2020, and July 31, 2020. The primary outcome was the diagnosis of COVID‐19, as confirmed by the real‐time reverse transcription‐polymerase chain reaction assay with nasal or pharyngeal swab specimens. Exposure variables are described in Table 1. This study was conducted in compliance with the Declaration of Helsinki. The institutional review board of Teine Keijinkai Medical Center approved the study (approval number 2‐020134‐00). Written informed consent for analyses and publication was obtained from all participants at the clinic visit.

**TABLE 1:** Demographic, clinical, and epidemiological characteristics of patients with and without coronavirus disease

	All (*n* = 315)	COVID‐19 patients (*n* = 27)	Non–COVID‐19 patients (*n* = 288)	*p*‐value
Age, years [25%, 75%]	60 [41, 74]	69 [37, 78]	52 [36, 73]	0.121
Women, *n* (%)	179 (57)	18 (67)	161 (56)	0.289
Hypertension, *n* (%)	46 (15)	4 (15)	42 (14)	0.980
Diabetes mellitus, *n* (%)	25 (8)	2 (7)	23 (8)	0.986
Chronic obstructive pulmonary disease, *n* (%)	4 (1)	1 (4)	3 (1)	0.239
Bronchial asthma, *n* (%)	18 (6)	0	18 (6)	0.239
Chronic kidney disease, *n* (%)	13 (5)	0	13 (5)	0.380
Coronary artery disease, *n* (%)	7 (2)	0	7 (2)	0.412
Heart failure, *n* (%)	4 (1)	0	4 (1)	0.537
Cerebral vascular disease, *n* (%)	6 (2)	1 (4)	5 (2)	0.477
Malignancy, *n* (%)	45 (14)	0	45 (14)	0.083
Body temperature, mean ± SD (℃)	36.9 ± 0.7	36.8 ± 0.7	37.0 ± 0.7	0.256
Mean systolic blood pressure, mean ± SD (mmHg)	100 ± 14	100 ± 9	100 ± 15	0.804
Pulse rate, mean ± SD (beats/min)	89 ± 17	90 ± 16	89 ± 17	0.734
Respiratory rate, mean ± SD (breaths/min)	19 ± 5	18 ± 4	19 ± 5	0.926
Oxygen saturation, mean ± SD (%)	97 ± 2	97 ± 2	97 ± 2	0.098
Fever^a^, *n* (%)	172 (55)	11 (41)	111 (56)	0.285
Cough, *n* (%)	134 (43)	16 (59)	118 (41)	0.068
Sore throat, *n* (%)	90 (29)	10 (37)	80 (28)	0.314
Headache, *n* (%)	80 (25)	9 (33)	71 (25)	0.327
Rhinorrhea, *n* (%)	61 (19)	8 (30)	53 (18)	0.161
Nausea or vomiting, *n* (%)	5 (2)	0	5 (2)	0.489
Abdominal pain or diarrhea, *n* (%)	45 (14)	3 (11)	42 (15)	0.617
Dysgeusia or anosmia, *n* (%)	28 (9)	4 (15)	24 (8)	0.261
Dyspnea, *n* (%)	73 (23)	6 (22)	67 (23)	0.895
Anorexia, *n* (%)	30 (10)	4 (15)	26 (9)	0.331
Fatigue, *n* (%)	132 (42)	16 (59)	116 (40)	0.058
Myalgia, *n* (%)	59 (19)	10 (37)	49 (17)	0.011
Dysuria, *n* (%)	11 (4)	2 (7)	9 (3)	0.248
Pneumonia^b^, *n* (%)	1 (0.3)	1 (4)	0	0.847
Fever^a^ within 2 preceding weeks, *n* (%)	247 (79)	19 (70)	228 (79)	0.271
Rhinorrhea or sore throat within 2 preceding weeks, *n* (%)	188 (60)	20 (74)	168 (59)	0.115
Fatigue within 2 preceding weeks, *n* (%)	148 (47)	14 (52)	134 (47)	0.607
Dysgeusia or anosmia within 2 preceding weeks, *n* (%)	30 (10)	4 (15)	26 (9)	0.331
Direct contact with COVID‐19 patients^c^ within 2 preceding weeks, *n* (%)	77 (25)	25 (93)	52 (18)	<0.001
Social gatherings^d^ within two preceding weeks, *n* (%)	47 (15)	10 (37)	37 (13)	0.031
Travel to areas with ongoing outbreaks of COVID‐19 within two preceding weeks, *n* (%)	3 (1)	0	3 (1)	0.593

Abbreviations: COVID‐19, coronavirus disease 2019; SD, standard deviation.

^a^ An axillary temperature of 37.0°C or higher.

^b^ A positive result of RT‐PCR for COVID‐19 and acute respiratory disorder characterized by at least one of the following signs and symptoms: (i) presence of cough or chest pain, (ii) respiratory rate >20 breaths per minute, and (iii) O_2_ saturation <94% with room air.

^c^ Participants who had been in direct contact with COVID‐19 patients for ≥15 min.

^d^ Singing activity at karaoke bars, business conferences, and family reunions.

## 3 RESULTS

A total of 315 patients visited the Teine Keijinkai Medical Center Infection Triage Clinic and underwent RT‐PCR tests, and COVID‐19 was diagnosed in 27 patients (8.6%). Compared to patients without COVID‐19, infected patients had a significantly higher percentage of having myalgia (37% vs. 17%); the infected patients were also more likely to have had direct contact with other COVID‐19 patients in the previous two weeks (93% vs. 18%). They were more likely to have participated in social gatherings in the previous two weeks (37% vs. 13%). Other clinical manifestations (ie, vital signs, fever, cough and dyspnea, preexisting comorbidity, and travel history to other prefectures) were comparable between the two groups. Among COVID‐19 patients, one patient required oxygen administration and the same patient was hospitalized for COVID‐19–associated pneumonia, treated successfully with dexamethasone without any sequelae. Other 26 patients did not require oxygen therapy and were instructed recuperation at home for seven to fourteen days. These 26 patients were returned to the daily life without the development of COVID‐19–associated pneumonia.

## 4 DISCUSSION

Our finding that patients with COVID‐19 had a significantly higher proportion of direct contact with confirmed COVID‐19 cases and participation in social gatherings in the two weeks before presenting at the clinic was consistent with previous studies.^3^, ^4^, ^8^ This may be biologically plausible because the main route of transmission of SARS‐CoV‐2 is through respiratory droplets.^9^ Additionally, our results showed that recent travel to areas with ongoing COVID‐19 outbreaks did not differ between the two groups. This may be because only three people had traveled to the endemic areas, and our analysis did not have sufficient statistical power to detect the difference. Moreover, we found that myalgia occurred with a significantly higher frequency among patients with COVID‐19 than those without the infection. Several case reports noted that myalgia was one of the most common symptoms in confirmed COVID‐19 cases.^10^ Other predominant symptoms included fever, cough, and dyspnea. A meta‐analysis assessing the clinical, laboratory, and imaging characteristics and outcomes of patients with COVID‐19 in 39 case reports and 19 observational studies reported that the frequency of fever, cough, and dyspnea was 89%, 58%, and 46%, respectively.^10^ In this study, the cough frequency was similar, but the frequencies of fever and dyspnea were lower than those reported in the meta‐analysis above. This can be attributed to the outpatient setting of this study; therefore, mild cases of COVID‐19 may have been more likely to present to our clinic. Notably, our study showed that a total of 30% of the patients with COVID‐19 had experienced dysgeusia or anosmia, which was a relatively lower frequency than that in previous reports.^10^ The reasons are unclear, but one possible explanation is that we may not have accurately captured the symptoms because we collected data based on the information from a self‐reported questionnaire.

This study has several limitations. First, our study has selection bias because this study was conducted in an outpatient clinic, and therefore, we may have missed patients with severe symptoms. Indeed, we had only one patient who required hospitalization for this disease. Second, there is a possibility of recall bias because information about symptoms two weeks before the clinic visit relied on a self‐reported questionnaire. Third, antigen testing using saliva or nasopharyngeal swabs was not used for COVID‐19 diagnosis because the test had not been introduced at our institution at the time of data collection. Undoubtedly, more effort should be made to continue capturing a comprehensive clinical picture of COVID‐19 patients in Japan. Nonetheless, our findings may be applicable to other clinical settings, such as emergency room, where performing a focused history taking is the cornerstone of diagnosis and treatment.

## 5 CONCLUSION

In conclusion, to our knowledge, this is the first report describing the clinico‐epidemiological characteristics of patients attending a specialized outpatient clinic for COVID‐19. More studies from Japan will be needed to contribute to the growing volume of clinical evidence and elucidate the complete clinical spectrum of the disease in the Japanese population.

## CONFLICT OF INTEREST

The authors have stated explicitly that there are no conflicts of interest in connection with this article.
